# A validation study on the accuracy and precision of gaze and vergence using stereoscopic eye-tracking technology

**DOI:** 10.3758/s13428-025-02731-1

**Published:** 2025-07-01

**Authors:** Arthur R. Pijpaert, H. H. L. M. Jeroen Goossens, Bob W. van Dijk, L. J. Bert Roetman, Ruth M. A. van Nispen, Laurentius J. René van Rijn

**Affiliations:** 1 Amsterdam UMC Location Vrije Universiteit Amsterdam, Ophthalmology, De Boelelaan 1117, Amsterdam, The Netherlands; 2 Amsterdam Neuroscience, Amsterdam, The Netherlands; 3 Donders Institute for Brain, Cognition and Behaviour, Department of Cognitive Neuroscience, Radboud University Medical Centre Nijmegen, Nijmegen, Netherlands; 4 Laméris Ootech B.V., Ede, Netherlands; 5 Amsterdam Public Health, Quality of Care, Amsterdam, The Netherlands

**Keywords:** Stereoscopic eye-tracking, Vergence measurement, Gaze accuracy, Pupil size, Gaze eccentricity

## Abstract

Binocular video-based eye-tracking allows for gaze and vergence measurements, but the accuracy and precision of vergence are barely addressed. Here, we investigate the accuracy and precision of both gaze and vergence measurements using a stereoscopic eye-tracking system. Previous studies have evaluated stereoscopic eye-trackers for gaze eccentricities up to 16°. We validated a custom-built stereoscopic eye-tracker with two cameras and two infrared light sources for gaze eccentricities up to 21°. Additionally, we studied the impact of fixation distance and pupil size on vergence accuracy. Participants with normal binocular vision (*N* = 8) performed fixation tasks, enabling the assessment of both gaze and vergence errors. The stereoscopic system provided gaze estimates with a mean absolute error (MAE) of less than 1° within the central visual field. However, the accuracy decreased for peripheral angles larger than 14°. We found a MAE of 0.89 ± 0.58° in measuring vergence and a strong linear association between target vergence and measured vergence, with a slope of 0.99 ± 0.05. In contrast to previous studies using single-camera eye-trackers, we found no systematic influence of pupil size on the vergence measurements. Although there was high agreement between estimated and ground truth vergence in the central field, the system did struggle to maintain accuracy at larger eccentricities. This limitation arises primarily from the loss of reliable glints rather than technical constraints, indicating the need for alternative approaches to enhance accuracy in wider fields of view.

**Supplementary Information:**

The online version contains supplementary material available at 10.3758/s13428-025-02731-1.

## Introduction

Eye-tracking technology has become integral in various fields ranging from psychology and neuroscience research to human–computer interaction and market research. Accurate and precise eye-tracking systems are essential for understanding human behavior, cognition, and visual attention.

Vergence is a critical aspect of eye movement behavior that has received less attention compared to other eye movements, despite its significance in various visual tasks (Jaschinski & Groner, 2020). Vergence is the simultaneous movement of both eyes in opposite direction to maintain or obtain binocular vision (Ciuffreda et al., 2014). In other words, vergence movements adjust the angle between the visual axes, aligning the foveae of both eyes with points of interest. The ability to focus on objects at different depths is important for many visual tasks; it enables high-resolution binocular vision and sensory fusion, which is essential for depth perception, and has important implications in attention research and eye alignment (Wong & Wong, 2008).

Exploring vergence more deeply can enhance our understanding of visual processing (Solé Puig et al., 2013). Better comprehension of vergence mechanisms can aid in developing new diagnostic tools and therapeutic strategies for conditions that affect binocular vision (Kapoula et al., 2016). Additionally, it can improve the precision and reliability of studies involving visual attention and depth perception (Solé Puig et al., 2013; Zhang, et al., 2024). By considering vergence, eye-tracking technology can become an even more powerful tool for a wide range of applications.

The majority of the currently available commercial eye-trackers use an image location of the pupil and one or multiple corneal reflections (glints) from one camera, together with a non-linear mapping function to estimate the gaze of the subjects. To obtain the mapping function, a calibration procedure has to be performed in which the subject has to look at multiple targets in the measurement range of the eye-tracker. A mathematical model suggests that using multiple cameras and multiple light sources can simplify the calibration procedure (Guestrin & Eizenman, 2006). This setup, known as a stereoscopic eye-tracker, reduces the calibration requirement to a single point to estimate the angle (often referred to as $\kappa$) between the optical axis and visual axis of the eye. The optical axis of the eye is a straight line passing through the center of the corneal curvature and the center of the entrance pupil, while the visual axis is the line connecting the center of the fovea with the fixation point through the nodal point of the eye. A minimal calibration procedure enables eye-tracking on humans with a lower attention span, such as young children and mentally disabled people (Barsingerhorn et al., 2019).

Several studies have shown that a stereoscopic eye-tracker can achieve accuracies of just above 1° (Zhu & Ji, 2007) or even below 1° (Barsingerhorn et al., 2018; Guestrin & Eizenman, 2008; Nagamatsu et al., 2008; Shih & Liu, 2004), in the estimation of gaze angles. Although these studies have shown acceptable accuracy, only horizontal gaze angles up to ± 16° were measured (Barsingerhorn et al., Dec 2018; Guestrin & Eizenman, 2008; Shih & Liu, 2004). These studies show that accuracy decreases for peripheral stimuli, suggesting it may be significantly worse than 1° for stimuli with an eccentricity greater than 16°.

Besides the limited range, in these studies, the performance of stereoscopic eye-trackers on vergence estimation was never evaluated. A previous study with standard single-camera eye-trackers has revealed that both gaze and vergence estimates obtained with such pupil-based binocular systems are significantly influenced by changes in the subject’s average pupil area (Hooge et al., 2019). Pupil dilation associated with normal adaptation to dark viewing conditions, for example, deviated the horizontal vergence estimates up to 2°. In addition, they found a linear association between measured change in vergence and pupil diameter of − 0.39 ± 0.09°/mm to − 0.72 ± 0.11°/mm. A simulation study (Wang et al., 2019) also showed that accuracy and precision limitations in gaze tracking yield a high error in depth when estimating the vergence point, i.e., the point where two lines of sight are closest to each other.

The goal of the present study is threefold. First, we aim to validate the accuracy and precision of our remote eye-tracker across a wide field of view. Second, we aim to evaluate the influence of pupil area on measured vergence using a stereoscopic eye-tracker. Finally, we aim to assess the performance of our system in estimating vergence angles.

## Materials and methods

The study was ethically approved by Amsterdam UMC. This study was performed according to the standards of the Declaration of Helsinki (1964) and its later amendments. Participants were recruited through flyers distributed within the ophthalmology department, targeting colleagues. All participants were healthy adults with normal visual acuity, without the use of glasses or contact lenses, and reported normal binocular vision. They had no known history of strabismus or other ocular conditions affecting vergence. The first experiment included six subjects (four female, 45 ± 14 years), and the second experiment included five subjects (four female, 35 ± 5 years). Three of our subjects participated in both experiments.

### Hardware

**Fig. 1:**
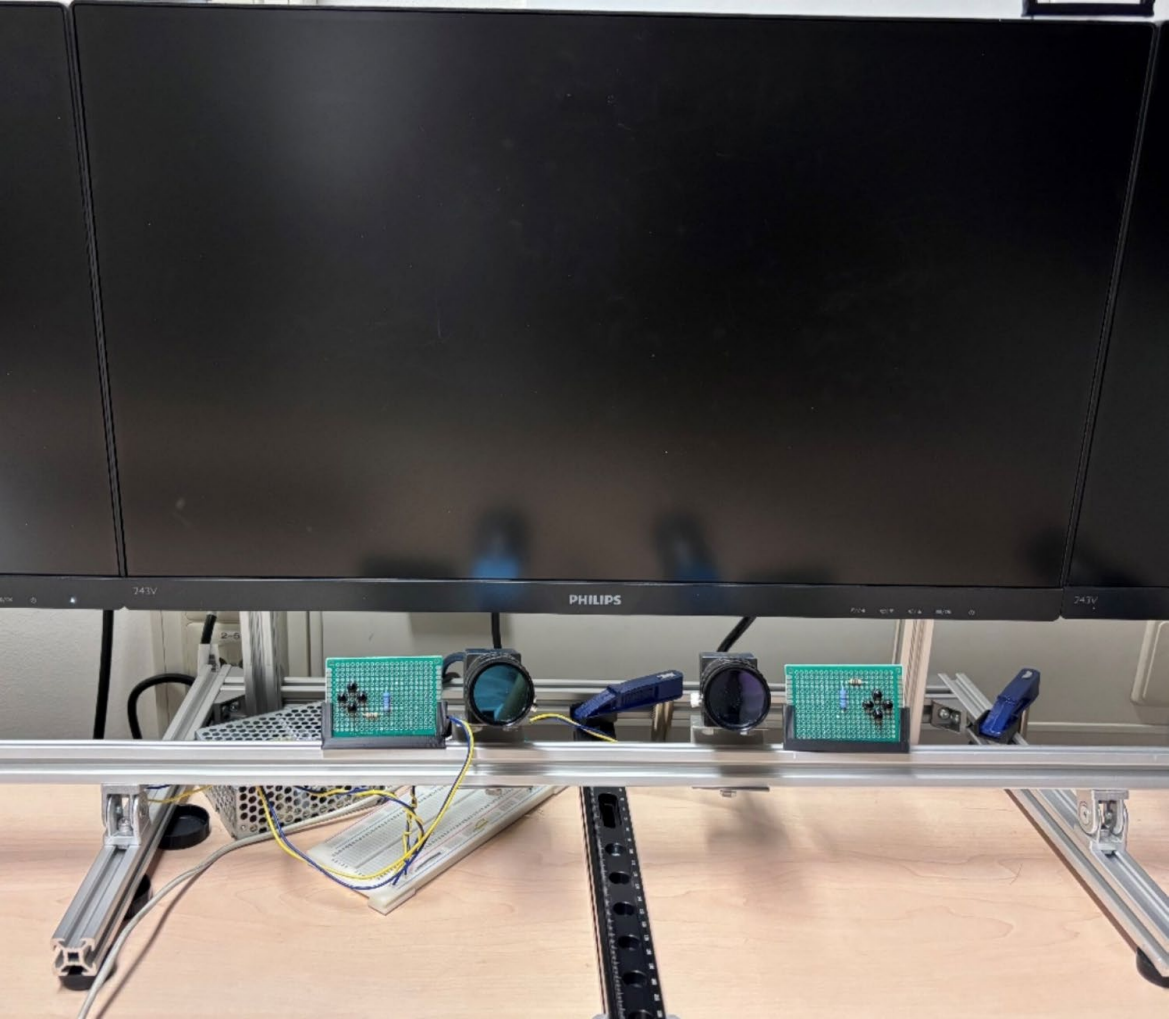
Stereoscopic eye-tracker hardware setup. *Note.* Two cameras with IR filters and two IR LED boards are mounted on an aluminum rail below a monitor. The cameras are positioned to capture both eyes for stereoscopic eye-tracking, with the LED boards producing glints on the subject*’*s eye and providing consistent illumination

The eye-tracker hardware consisted of two cameras, two infrared light sources, and a monitor (Fig. 1). The cameras (MER2-630-60U3M, Daheng Imaging, Beijing, China, 2.4 × 2.4 μm) and light sources (each with four 850-nm LEDs, OSRAM Radial T1 3/4, SFH 4557, ams-OSRAM AG, Premstätten, Austria) were positioned on a desk, 150 mm below and 40 mm in front of the monitor (Philips 243V7QDAB, Royal Philips, Amsterdam, The Netherlands). The cameras were placed 60 mm to the left and right of the screen’s center, while the light sources were positioned an additional 60 mm further out. The manual focusing lenses had a diaphragm and a focal length of 16 mm (LCM-5MP-16MM-F1.4–1.5-ND1, Daheng Imaging, Beijing, China). Visible light was blocked by infrared-passing filters (LFT-BP830-M30.5, Daheng Imaging, Beijing, China) with a transmission band between 802 and 868 nm.

The software of the stereo eye-tracker was operated on a desktop PC equipped with a sixteen-core CPU with a base frequency of 3.4 GHz (Intel® Core i7-13700 K, 3,4 GHz, Santa Clara, CA, USA), a 12-GB graphics card (GIGABYTE GeForce RTX 4070 Ti AERO OC 12G, Taipei, Taiwan) and Ubuntu 22.4 (Ubuntu, London, England) as operating system. Due to the high default resolution of the cameras (3088 × 2064 pixels), image processing was very time-consuming. To reduce the load, a region of interest (ROI) of 1420 × 1420 pixels was used around the center of the camera sensor.

### System calibration

The system was calibrated to retrieve the intrinsic and extrinsic parameters of the cameras, the location of the light sources, and the position and orientation of the screen, following the procedure of Barsingerhorn (Barsingerhorn et al., 2018). First, the intrinsic and extrinsic parameters of the stereo camera pair were determined using camera calibration functions from OpenCV (Bradski, 2000). This was done by taking a series of pictures of a planar checkerboard pattern (7 × 10 grid, 15-mm square size) with both cameras simultaneously. First, the checkerboard images were used to determine the intrinsic parameters (focal points, optical centers, and distortion coefficients) of each camera. Subsequently, a stereo calibration was performed using the acquired stereo image pairs to estimate the extrinsic parameters. The latter specifies the rotation and translation that transform a point from the coordinate system of the second camera to the coordinate system of the first camera. This rotation is expressed in the coordinate system of the first camera.

It is not possible to determine the location of the light sources and the pose of the monitor directly because they are behind the cameras’ line of sight. Therefore, a planar mirror was used to get virtual stereo images of the light sources and the monitor, as in previous studies (Barsingerhorn et al., 2018; Beymer et al., 2003; Chen et al., 2008; Shih & Liu, 2004; Zhu & Ji, 2007). The pose of this planar mirror was estimated from a dot pattern on its reflecting surface. Virtual images of the light sources were located in 3D, and the law of reflection was applied to calculate the true 3D positions of the light sources relative to camera 1 of the stereo pair. A dot pattern was also attached to the monitor so that the center and pose of the monitor could be determined from the virtual images of the dot pattern using triangulation and the law of reflection. Care was taken to align the center and orientation of the dot pattern with the center and (in plane) orientation of the screen.

### Software

Eye-tracker software was developed in Visual Studio Code using Python 3.10.12 (Python Software Foundation, www.python.org). To acquire frames and set acquisition parameters on the cameras, the GxIAPI software development kit (Daheng Imaging, Beijing, China) was used. The main task of the software was to acquire frames from the cameras and perform image processing to determine the image location of the centers of the pupils and glints in the camera frames. The experimenter could adjust feature-detection parameters through the Graphical User Interface (GUI, see supplement A). These parameters—such as the threshold and size for detecting pupils and glints, as well as histogram equalization—were applied to enhance the image for more accurate detection of pupils and glints.

A Python algorithm was developed to extract pupil and glint data. A flow diagram of the algorithm can be found in supplement B. The algorithm first located the eyes using a face detection model from Mediapipe (Google, Mountain View, CA, USA). This location was then used to set an ROI of 200 × 200 pixels around each eye.

Within the ROI, the pixel coordinates of the pupil and glints were estimated. The ROI was binarized using a threshold set in the GUI, and the pupil candidates were detected using a contour finding algorithm. The pupil candidates were filtered based on the pupil area in pixels. From the remaining pupil candidates, the candidate with the largest pupil area was taken as the pupil.

An ellipse was fit on the pupil contour. The center of the ellipse was used to describe the position of the pupil, and the area of the ellipse as the area of the pupil. Information about the pupil center and the area of the pupil in pixels was subsequently updated.

If a pupil was found, glint candidates were detected using thresholding with the values set in the GUI. These glints were also filtered based on their pixel area. The centroid of the glint pixels was used as the position of the glint. Glints were not expected above the pupil image center due to the setup’s geometry, as the subject’s gaze never extended below the light sources. Therefore, any glints appearing in that region were removed during data processing.

Among the remaining glint candidates, the two glints closest to the pupil in pixel coordinates were taken as the glints. Finally, the last two glints were sorted on their horizontal (*x*-axis) location; the glint with lower *x*-value was assumed to be glint 1 and the glint with a higher *x*-value was assumed to be glint 2 corresponding to the reflection of the light source one and two on the cornea, respectively.

Information about the position of the glints was updated and the data about the pupils and glints were saved with a corresponding timestamp. If no pupil and/or glint was found, NaN-values were stored instead.

Online feedback was given through plots of the pupil-glint vectors as a function of time in the GUI. If something went wrong during measurement, it could be seen in these plots. This feedback did not display the actual gaze of the participant, but it did show uncalibrated movements of the eye. Besides the eye-tracking functions of the software, it was also programmed to present visual stimuli. In the configuration file, the monitor for stimulus presentation could be chosen. When the task was started, the stimuli were shown on the stimulus window as well as on the lower left part of the tracker application. The stimulus window was shown on a separate monitor in front of the participant.

### Gaze reconstruction

Gaze reconstruction was performed offline. The reconstruction procedure was adapted from previously described methods [https://github.com/Donders-Institute/Stereo-gaze-tracking.git] (Barsingerhorn et al., 2018). The cameras operated at a frame rate of 50 Hz and were synchronized using software triggers to capture frames simultaneously. Despite this synchronization method, a small timing discrepancy of 0.1 s was detected in the recorded timestamps. This discrepancy was identified by comparing the frame timestamps of simultaneously captured images between the two cameras. To adjust for this small asynchrony in the image acquisition, the data from the second camera were interpolated to the timestamps of the first camera.

**Fig. 2:**
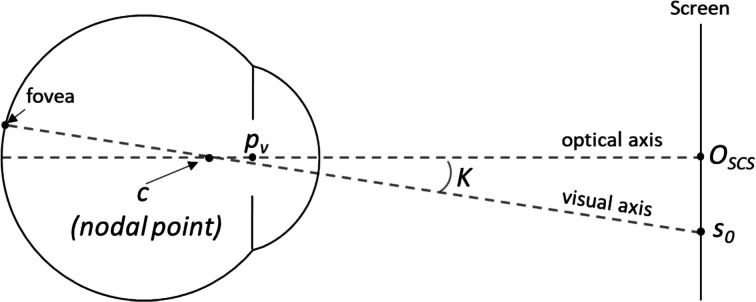
Visual representation of the eye’s optical and visual axis during calibration. Not drawn to scale. *c:* Center of corneal curvature. *p*_*v*_: virtual pupil. $\kappa$: deviation angle. *O*_*scs*_: origin of the screen coordinate system. *s*_*0*_: fixation point

The gaze reconstruction scripts [Stereo-gaze-tracking/gaze-reconstruction at master · Donders-Institute/Stereo-gaze-tracking · GitHub] return the spatial position of the corneal center of curvature (defined as the center of the best-fit spherical approximation to the corneal surface, $c$) and the center of the virtual pupil (${p}_{v}$), and are translated and rotated in the right-handed Cartesian coordinate system of the screen (O_SCS_). The optical axis was defined as the line that passes through both $c$ and ${p}_{v}$. The deviation angle ($\kappa$), which has a horizontal and vertical component, is determined through subject-specific calibration with a single fixation point (${s}_{0}$), as indicated in Fig. 2. Once $\kappa$ is determined, the visual axis can be calculated from the measured the optical axis. The point of gaze (POG) was defined as the point at which the visual axis intersects with the stimulus screen. To improve the precision of the POG estimates, we used the notion that the distance between $c$ and ${p}_{v}$ remains fixed (Barsingerhorn et al., Dec 2018;  Chen et al., 2008).

### Experiments

Prior to the experiments, the subjects performed a monocular calibration for each eye. During the calibration of one eye, the participant had to fixate a single target (a black circle of 0.5 degrees in diameter on a gray background [94.6 cd/m^2^] (Barsingerhorn et al., Dec 2018)) at the center of the screen while the other eye was occluded (Barsingerhorn et al., 2018). This monocular calibration ensured that estimation of the eye-specific angles $\kappa$ were unaffected by fixation disparity, a physiological misalignment of the eyes with the target that occurs naturally under binocular viewing conditions (Švede et al., 2015).

#### Experiment 1

The goal of the first experiment was to assess the accuracy and precision of the stereo eye-tracker. For this experiment, the subject had to fixate on multiple visual stimuli that appeared one after another on a screen. During the experiment, the positions of the pupil and glints were measured, and after each measurement, translated to gaze angles. From these gaze angles, the accuracy and precision of the gaze were determined, as well as the vergence error.

The distance between the subjects and the screen was 650 mm, with a cyclopean eye located on the *z*-axis of the screen. The participants'heads were fixated using a bite board to reduce unwanted head movements. This is important during validation because the calculations of accuracy assume a fixed position of the eyes relative to the visual stimuli. For other applications, head fixation is not needed for this particular setup (Guestrin & Eizenman, 2006).

The fixation targets (same 0.5 degree-targets as used for calibration) were presented at different locations for 2 s in a pseudo-random order with an inter-target interval ranging from 1 to 1.4 s. The targets were presented at horizontal positions ranging from – 21° to 21° in increments of 7°, and at vertical positions ranging from – 10° to 10° in increments of 5°, with the center of the screen as the origin. Each target position was presented once. The task was performed under both binocular and monocular viewing conditions, with gaze calculated separately for the left and right eyes in all conditions, totaling three tasks per participant.

We used visual inspection of the data to determine the fixation window, by closely examining the raw fixation data.

#### Experiment 2

We further evaluated the performance of measuring vergence with our stereoscopic eye-tracker by testing the influence of changing vergence angles and the projected pupil area, as seen by the camera, on the measured vergence. Towards that end, we asked our subjects to perform two tasks.

In the first task, the subject was instructed to fixate on one of five targets on a wire frame (see Supplement C), each for 2 s, in a pseudo-random order. This task was performed for three different viewing distances of the target frame to the subject. The experimenter manually adjusted the position of the frame, inducing a change in vergence for all five targets. The central target was placed at 610, 480, and 350 mm from the subjects’ cyclopean eye.

For the second task, the subject was instructed to focus on a target positioned at the center of the screen. The experiment was conducted in a dark environment, with ambient lighting systematically manipulated by adjusting the brightness of two screens positioned next to the stimulus presentation screen. The ambient lighting ranged from 4.06 lx to 35.12 lx, achieved by incrementally increasing the brightness of these screens in five steps. The luminance of the fixation target and the background luminance of the stimulus screen were kept the same compared to experiment 1. Note, that viewing distance was kept constant in this task to avoid changes in pupil size in relation to changes in true vergence as part of the near triad response.

### Analysis

The first 0.6 s after stimulus onset were excluded from the analysis to account for potential saccades. Similarly, the last 0.5 s of stimulus presentation were excluded to avoid capturing anticipatory effects or preparatory responses as participants could potentially begin to prepare for the next target. The remaining data points define the fixation window, ensuring stable target fixation for analysis. For each stimulus position, one fixation window was used for analysis.

Fixation windows identified as outliers were excluded from the analyses. A fixation window was identified as outlier when the standard deviation of the *x*- and or *y*-signal of the gaze angles during the fixation window was higher than 2 degrees, as this indicated large eye movements or blinks that disrupt stable fixation.

### Fixation

For the analysis of the fixation, the point of gaze was converted from screen coordinates in mm to gaze angles ($g$) in degrees. These gaze estimations indicate the orientation of a theoretical eye placed on the *z*-axis of the screen coordinate system at 650 mm from the screen center and directed towards the same point on the screen that was observed by the tracked eye.

For each target the mean fixation location was calculated from the fixation window.

The horizontal and vertical accuracy of fixation were assessed using the mean absolute error (MAE_gaze_) of the estimated gaze relative to the stimulus location

1 $$MA{E}_{gaze}=\frac{\sum_{i=1}^{n}{|p}_{stim,i}-{g}_{i}|}{n}$$

where ${p}_{stim}$ is the position of the stimulus (in deg), the gaze angles ${g}_{i}$ (in deg), and $n$ is the number of datapoints included in the fixation window.

Precision was assessed using two metrics: the standard deviation (SD) and the sample-to sample root mean square angular displacement (S2S) (Holmqvist et al., 2012; Wang et al., 2017). The $SD$ for a set of $n$ samples, ${g}_{1}, \dots {g}_{n}$ was calculated as

2 $$SD=\sqrt{\frac{1}{n}\sum_{i=1}^{n}{\left({g}_{i}-\widehat{g}\right)}^{2}}$$

where $\widehat{g}$ is the mean fixation position. This was calculated for the horizontal ($x$) and vertical ($y$) components separately. The vectorial precision was calculated using the bivariate contour ellipse area (BCEA) instead of the 2D standard deviation, defined in Wang et al. (2017); Holmqvist et al., 2012), because the latter does not account for the correlation between the horizontal and vertical components of gaze. BCEA (in deg^2^) was calculated as (Guadron et al., 2022)

3 $$BCEA=2k\pi {SD}_{x}{SD}_{y}\sqrt{(1-{\rho }^{2})}$$

where ${SD}_{x}$ and ${SD}_{y}$ are the standard deviations along the horizontal and vertical meridians, respectively. The term $\rho$ represents the product–moment correlation between the two position components (Steinman, 1965). The value $k$ was set to 1.14, corresponding to a 68.3% probability that a given observation falls within the elliptical area.

The S2S precision was calculated as

4 $$S2S=\sqrt{\frac{1}{n-1}\sum_{i=1}^{n-1}{\theta }_{i}^{2}}$$

with ${\theta }_{i}$ being the vectorial distance between two subsequent samples $i$ and $i$+1

5 $${\theta }_{i}=\sqrt{{\left({x}_{i}-{x}_{i+1}\right)}^{2}+{\left({y}_{i}-{y}_{i+1}\right)}^{2}}$$

A distinction was made between all stimuli, stimuli within a horizontal range of – 14 to 14 degrees (central stimuli) and stimuli outside the range of – 14 and 14 degrees horizontally (eccentric stimuli). This distinction was made to find out if the accuracy and precision of the gaze measurements decreased for the eccentric viewing angles.

### Vergence

To calculate target vergence (${\alpha }_{target}$), both the viewing distance ($d$) and the interocular distance (IOD) is needed. IOD is the distance between the center of rotation (COR) of the left and right eye. To determine the COR we assumed that the radius of the corneal curvature ($r$) was 7.72 mm (Navarro et al., 1985) and the COR lay 13.5 mm behind the vertex of the cornea (Gross et al., 2008). From the eye-tracker data, we obtained the center of corneal curvature (c) and the eye’s optical axis (OA), which is a unit vector describing its orientation. This resulted in the COR being at:

6 $$COR=c-\left(13.5-r\right)OA$$

The IOD is then computed as

7 $$IOD=\Vert CO{R}_{left}-CO{R}_{right}\Vert$$

where $CO{R}_{left}$ and $CO{R}_{right}$ are the centers of rotation of the left and right eye, respectively. With the location of the $COR$ s of both eyes known, the distance of both the left (${d}_{left}$) and right eye (${d}_{right}$) to the target location $(t)$ is given by

8a $${d}_{left}=\Vert CO{R}_{left}-t\Vert$$

8b $${d}_{right}=\Vert CO{R}_{right}-t\Vert$$

The target and the two eyes formed a triangle of which the target vergence angle (${\alpha }_{target}$) could be calculated using the cosine law:

9 $${\alpha }_{target}={\text{cos}}^{-1}\left(\frac{{d}_{left}^{2}+{d}_{right}^{2}-IO{D}^{2}}{2{d}_{left}{d}_{right}}\right)$$

If both eyes fixated perfectly, the visual axis of the left eye should intersect with the visual axis of the right eye at the target location. However, this often was not the case. The measured vergence (${\alpha }_{measured}$) was taken as the angle between the visual axis ($VA$) of both eyes (estimated during gaze reconstruction). This was achieved by using the inverse cosine formula of the two vectors

10 $${\alpha }_{measured}={\text{cos}}^{-1}\left(\frac{V{A}_{left} \bullet V{A}_{rigt}}{\left|V{A}_{left}\right|\left|V{A}_{right}\right|}\right)$$

Here ${\alpha }_{measured}$ was the combined horizontal and vertical vergence and was averaged per fixation window.

To estimate the accuracy of the vergence measurements, we computed the mean absolute error of the measured vergence relative to target vergence

11 $$MA{E}_{vergence}=\frac{\sum_{i=1}^{n}\left|{\alpha }_{measured,i}-{\alpha }_{target,i}\right|}{n}$$

In addition, we examined the associations between target vergence and measured vergence, as well as between measured projected pupil area and measured vergence using linear mixed effects regression analyses in MATLAB R2022b. These analyses allowed us to account for both systematic (fixed) and subject-specific (random offset) effects, providing a more robust understanding of the relationships. More specifically, we assumed that the random effects capture the subject-specific fixation disparity while the fixed effects capture the vergence recording performance of the stereo eye-tracker (first task) or systematic errors induced by changes in pupil area (second task).

Furthermore, Spearman’s rank correlation tested the relationship between average pupil area and measured vergence using the second task.

### Comparison of monocular and binocular viewing conditions

A key assumption underlying the above-defined accuracy measures for the eye-tracker is that participants precisely foveate the target. Since this condition is not necessarily met under binocular viewing conditions due to fixation disparity, we aimed to determine whether the accuracy estimates obtained under binocular viewing conditions were comparable to those obtained under monocular viewing. To assess whether there were significant differences between the two methods, paired *t* tests were performed comparing the mean fixation accuracy obtained under the two condition.

## Results

### Raw data

**Fig. 3:**
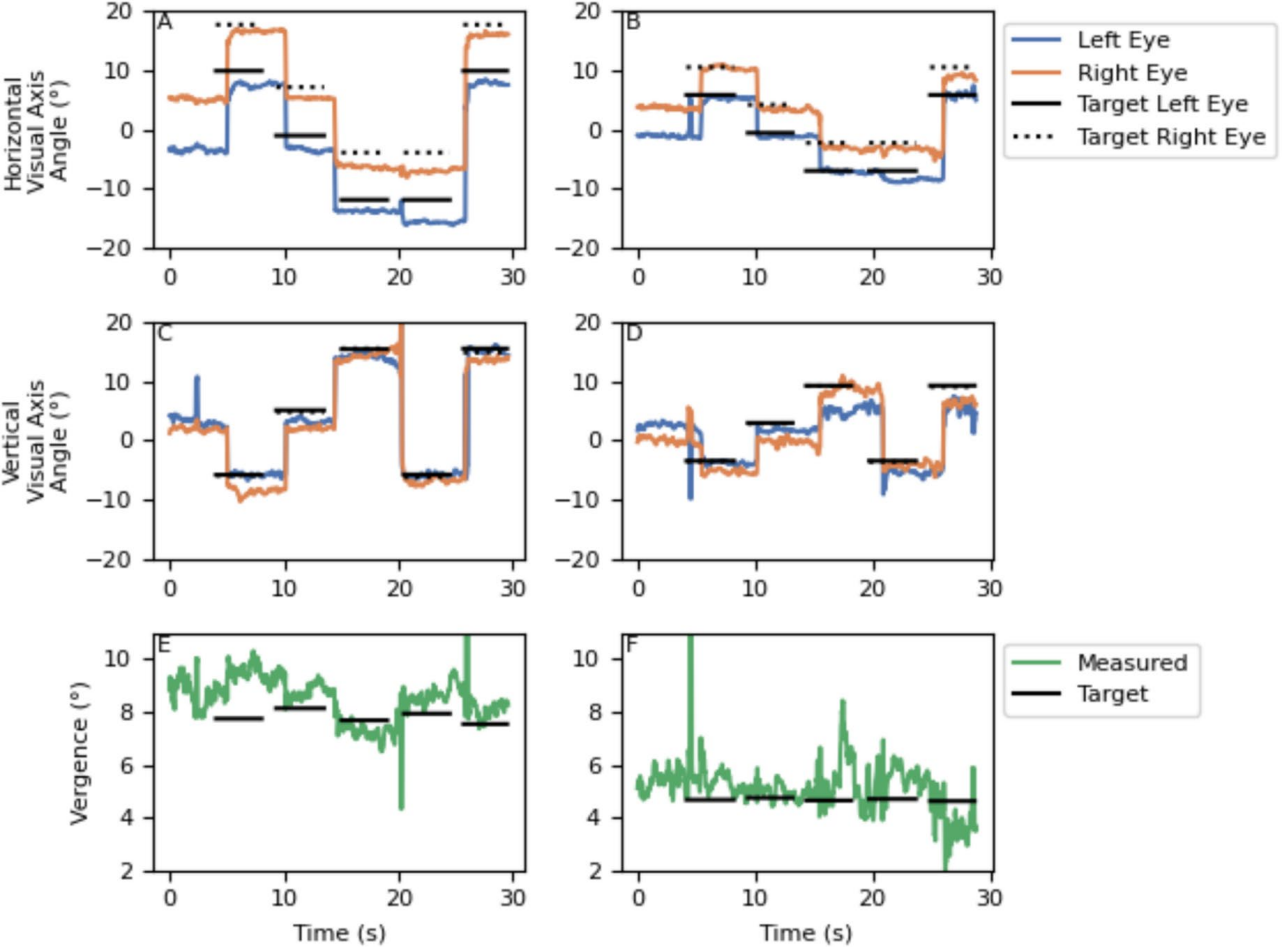
Horizontal and vertical visual axis angles and the measured vergence over time for a single subject. *Note.* **A**, **C**, **E** *D*ata measured with targets at 350 mm from the subject*,* **B**, **D**, **F** *D*ata with targets at 610 mm. The dotted target vergence line for the right eye in plots C and D overlaps with the solid target vergence line for the left eye due to the absence of vertical target vergence

The left-hand and right-hand panels of Fig. 3 show the visual angles of both eyes and the horizontal vergence in one of our participant while the subject looked at near (350 mm) and far (610 mm) targets, respectively. The data are taken from the second experiment. As expected, the horizontal angles of the left and right eye were more dissimilar at 350 mm (left-hand panels) than at 610 mm (right-hand panels) viewing distance. The vertical angles for both eyes and both distances were similar. The measured horizontal vergence for the closer targets was greater than the horizontal vergence for the distant stimuli.

#### Experiment 1

**Fig. 4:**
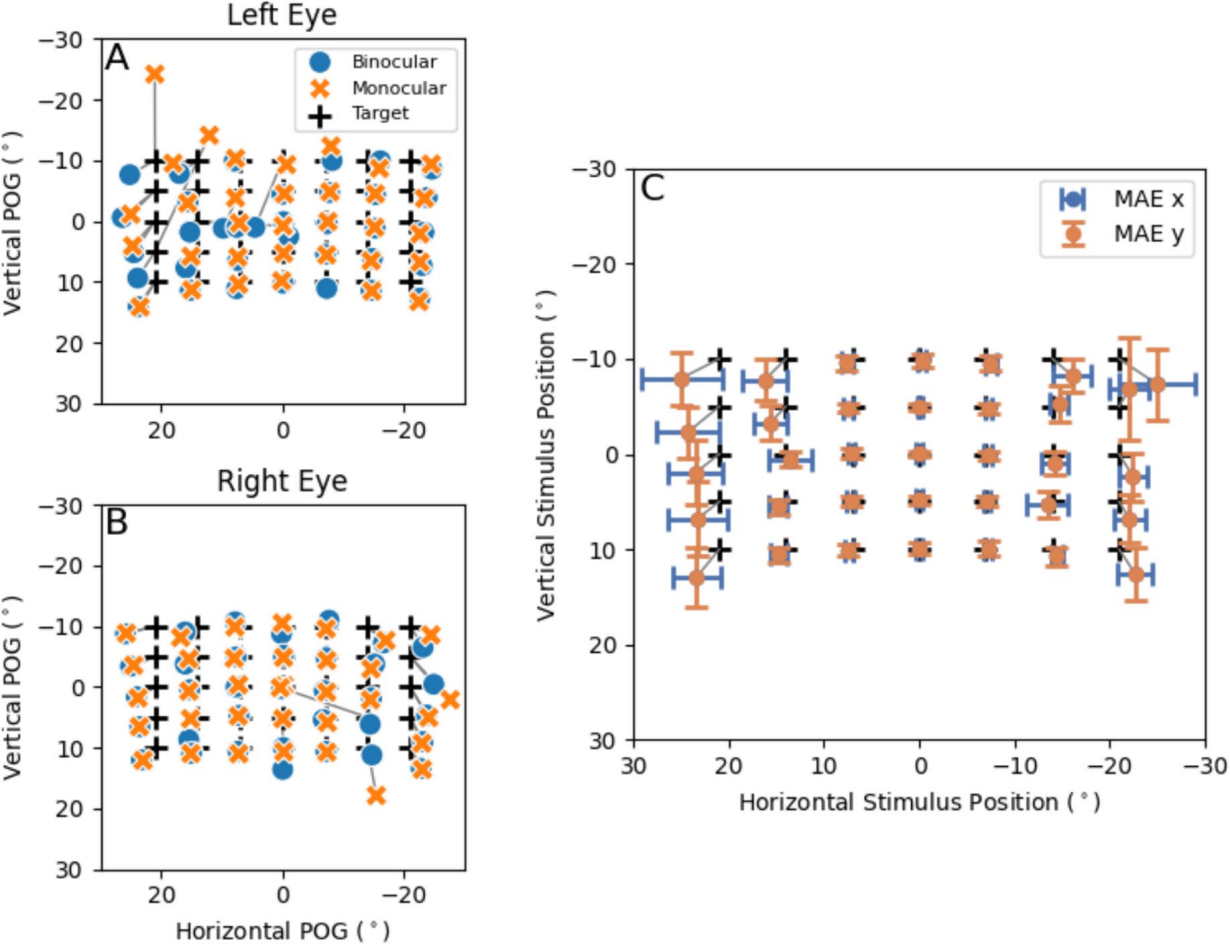
Gaze angle estimations of one subject and mean absolute errors (MAEs) expressed as error bars. *Note.* **A**, **B** show gaze angle estimations of one subject from monocular and binocular measurements for the left and right eye, respectively. **C** MAE expressed in error bars for each stimulus location, plotted on the mean fixation location. Due to the right-handed coordinate system used in the setup, positive angles are to the left and down. In **C**, the error bars show the horizontal (MAE x) and vertical (MAE y) mean absolute error averaged across all eyes, subjects and viewing condition. The black crosses in the figure represent target locations

**Fig. 5:**
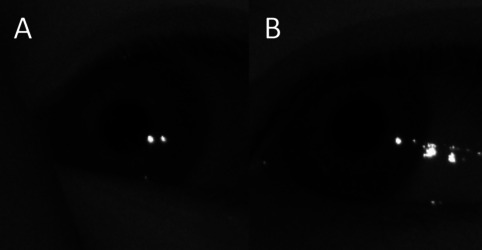
Example of glints in eye images. *Note*. **A** Example of two well-detectable glints on the corneal surface.** B** Example where one glint remains well-detectable, while the other is near the cornea-sclera border, appearing smaller and more scattered due to optical distortion

In the first experiment, the subjects had to fixate several visual stimuli presented on the planar screen positioned at a fixed distance. Initially, they used monocular vision, with their left eye first and then their right eye. Subsequently, they viewed the same visual stimuli with both eyes. For this first experiment, 14% of the fixation windows were identified as outliers, which we removed before further analysis (Methods). Monocular (dots) and binocular (crosses) gaze estimates for one participant are shown in Fig. 4A and B. Gaze estimates for angles up to 7 degrees left and right and 10 degrees up and down were very close to the target locations (plusses). The gaze estimates differed slightly from the target for the targets at a 14 degrees positive and negative horizontal angle, especially for the targets at the top (– 10° vertically). For the most eccentric horizontal targets (21 degrees positive and negative horizontally), gaze estimates were less accurate. This decrease in accuracy for peripheral targets may be attributed to optical distortions of glints as they move beyond the spherical region of the cornea at eccentric viewing directions. Figure 5A shows an eye image from one of the camera’s with two well-detectable glints during central viewing, while Fig. 5B shows how glints appear smaller (sometimes making them undetectable) and more scattered, both leading to reduced tracking accuracy during eccentric viewing. The decreased accuracy is further demonstrated by Fig. 4C***,*** which shows the average MAE for each stimulus position pooled for both monocular and binocular measurements over both eyes of all subjects.

**Fig. 6:**
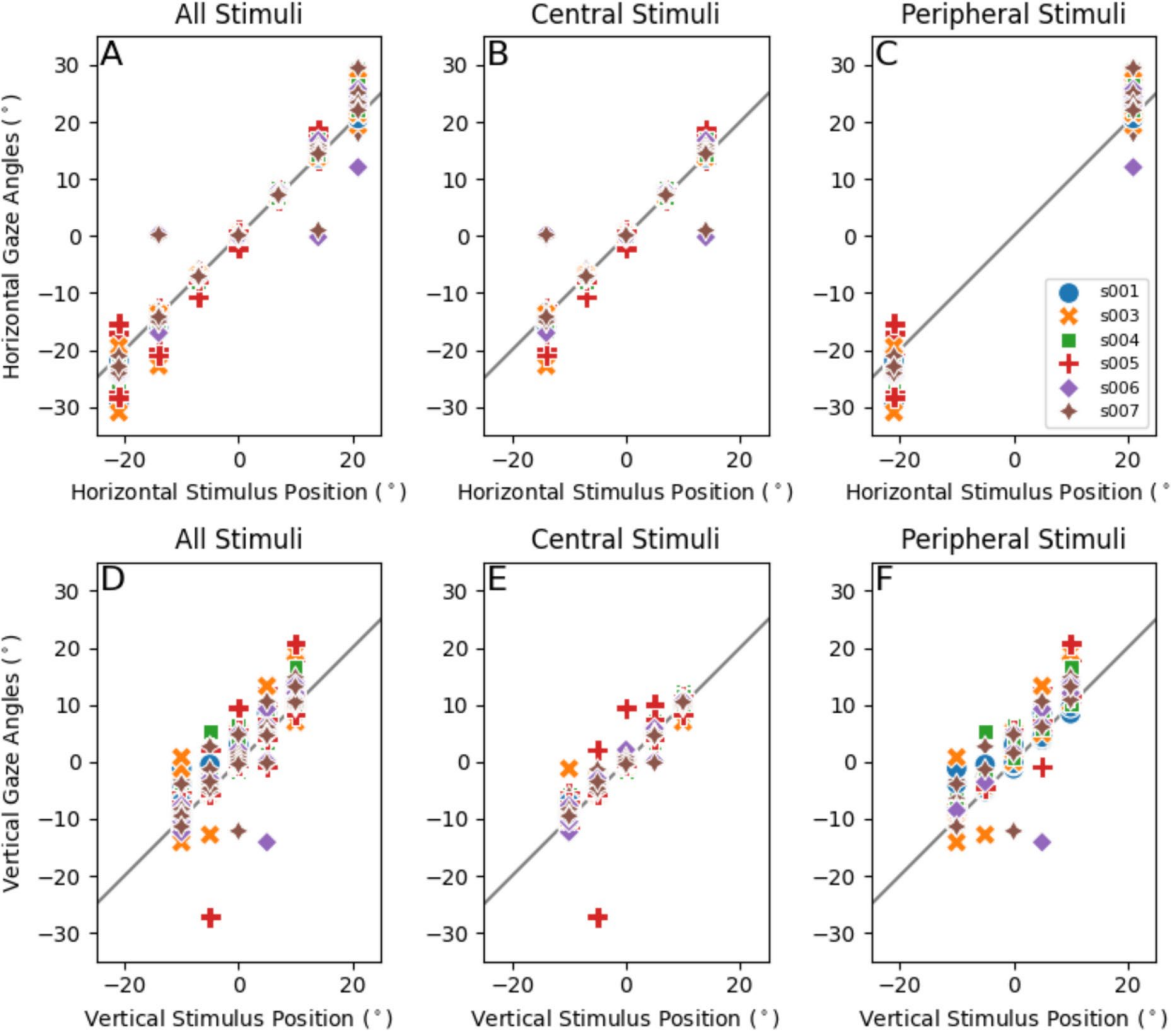
Horizontal and vertical gaze estimates against target position. *Note.* Each point is an averaged gaze estimation for each fixation trial. It shows data for each fixation, eye, viewing condition and subject. **A***,* **B** The horizontal gaze angles versus de horizontal stimulus position. **C***,* **D** The vertical gaze angles versus the vertical stimulus position. **B***,* **D** *S*timuli between *– *14° and 14° horizontallyonly

The scatter plots in Fig. 6 show the horizontal (A, B, C) and vertical (D, E, F) gaze angles of our subjects as a function of stimulus position. Figure 6A and D show the fixation data for all targets whereas Figs. 6B and E only show fixations for stimuli between – 14° and 14° horizontally. Figure 6C and F show fixations for the peripheral targets. The solid lines are identity lines. Note that the gaze estimates for central target locations were closer to the target, while deviations were greater for peripheral targets. Furthermore, vertical errors were larger when fixating on targets with a large horizontal component compared to those with a small horizontal component. When isolating the stimulus points between – 14 and 14 degrees horizontally, there were fewer large errors (Fig. 6B). Figure 6C and F show that errors for peripheral targets are larger relative to the central stimuli.

**Fig. 7:**
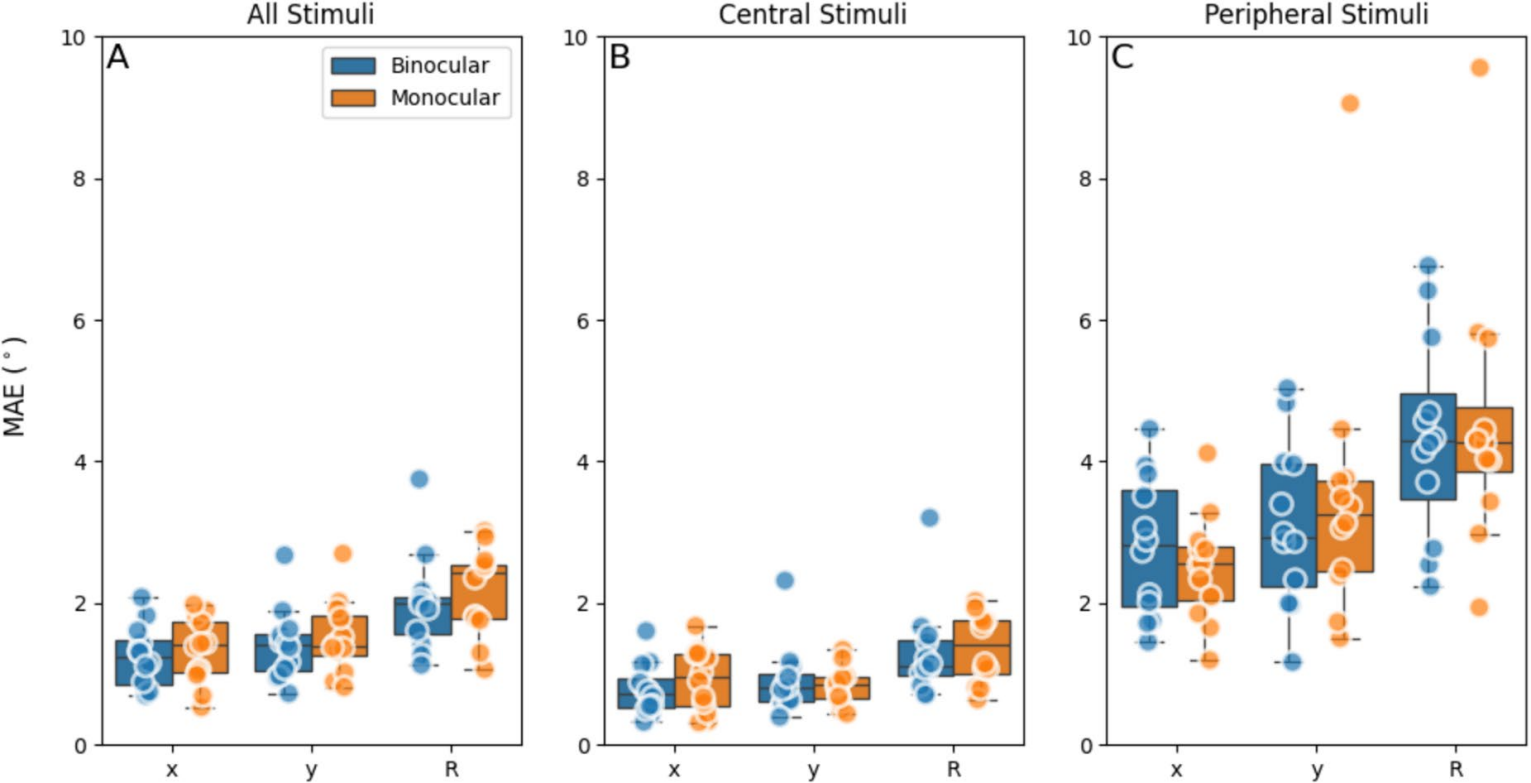
Box plots showing mean absolute error (MAE_gaze_) accuracy of the eye-tracker in estimating gaze for the horizontal (*x*), vertical (*y*), and vectorial (r) components under monocular and binocular viewing conditions. *Note. ***A **Data including all stimuli positions. **B **Only stimuli positioned between – 14° and 14° horizontally. **C **The stimuli outside of – 14° and 14° horizontally. Dots represent individual subject data. MAE of each individual subject was calculated by averaging the MAEs of both eyes across all (selected) stimulus positions

We performed measurements under monocular and binocular viewing conditions to evaluate potential differences in performance assessments between the two viewing conditions. Figure 7 presents the accuracy data, showing better accuracy for the central stimuli, and a decreased accuracy for gaze at eccentric stimuli. This observation is supported by the mean values of MAE displayed in Table 1. The table indicates that the mean of MAE is higher for all stimuli compared to the central stimuli, for both monocular and binocular viewing conditions, due to the large errors at eccentric locations. However, paired t-tests did not reveal any significant differences between the monocular and the binocular viewing condition.

**Table 1:** Average accuracy expressed in mean absolute error (MAE_gaze_) of the horizontal, vertical. and vectorial component of the gaze

MAE_gaze_	Mean_Monocular_ ± SD (°)	Mean_Binocular_ ± SD (°)	*t*-value	*p* value
All stimuli				
* x*	1.33 ± 0.47	1.24 ± 0.45	−0.48	0.63
* y*	1.51 ± 0.53	1.41 ± 0.52	−0.48	0.63
r	2.18 ± 0.62	2.00 ± 0.70	−0.65	0.52
Central stimuli				
* x*	0.93 ± 0.45	0.78 ± 0.37	−0.87	0.39
* y*	0.83 ± 0.45	0.90 ± 0.50	0.41	0.68
r	1.37 ± 0.50	1.31 ± 0.67	−0.26	0.79
Peripheral stimuli				
* x*	2.50 ± 0.77	2.79 ± 1.00	0.79	0.44
* y*	3.51 ± 1.94	3.11 ± 1.18	−0.60	0.55
R	4.57 ± 1.89	4.35 ± 1.44	−0.32	0.75

**Fig. 8:**
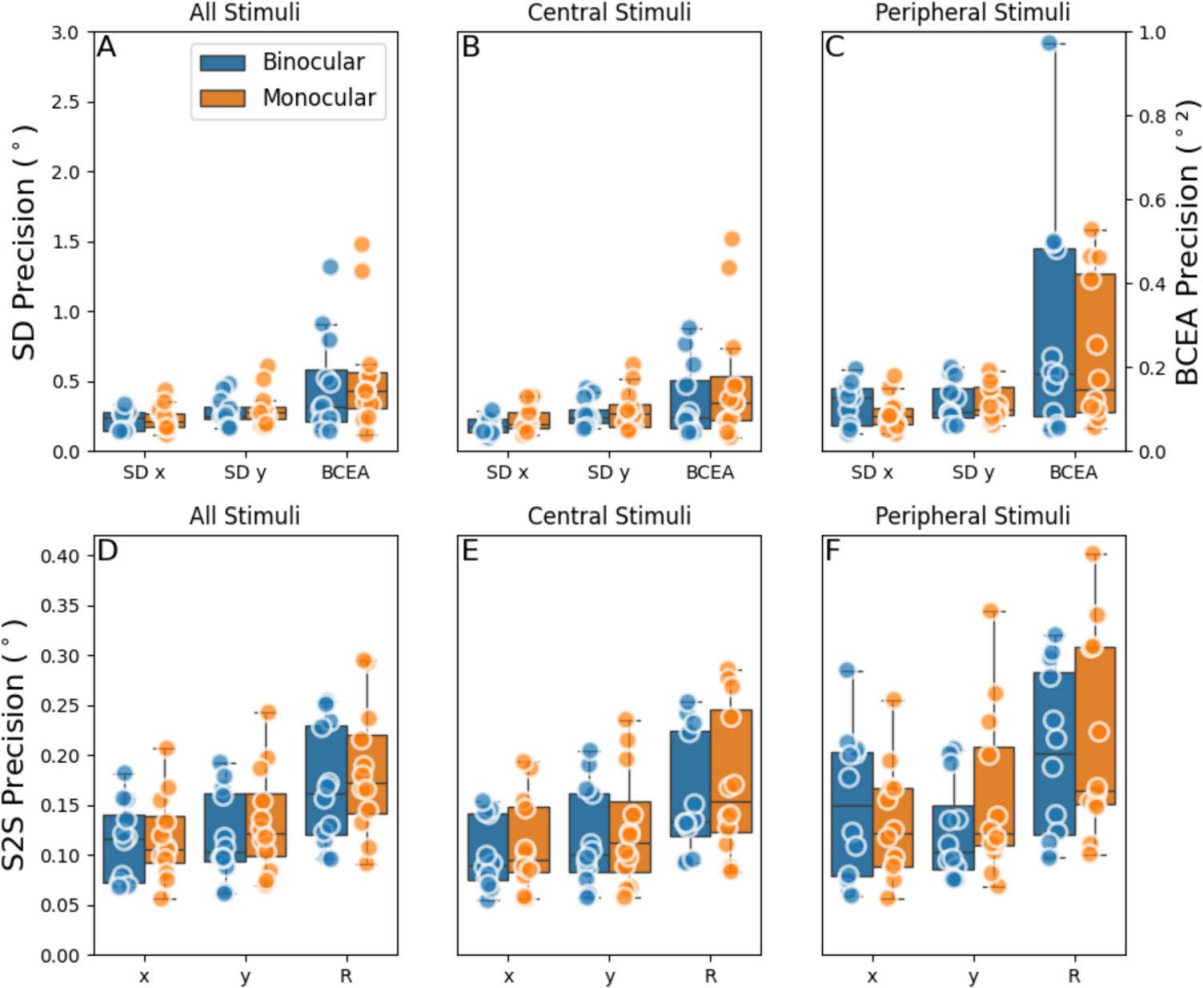
Box plots showing precision of the eye-tracker. *Note.* Precision is shown for the horizontal (SD x, S2S x) vertical (SD y, S2S y) and vectorial (S2S R) components and bivariate contour ellipse area (BCEA) vectorial precision for monocular and binocular measurements. **A**, **D** Data including all stimuli positions. **B**, **E** Only stimuli position between – 14° and 14° horizontally. **C**, **F** The stimuli outside of – 14° and 14° horizontally. For each subject, SD and BCEA precision measures are calculated by averaging the respective precision measure across all (selected) stimulus positions and both eyes. The left vertical axis shows the values for SD (in deg), where the right vertical axis shows the values for BCEA (in deg^2^)

Figure 8A, B, and C show the precision expressed per subject as SD and BCEA. Note, that the precision was higher when only the central stimuli were considered. The same can be seen in Fig. 8D, E and F, which plot the S2S precision. The mean precision measures are summarized in Table 2. These results also show a significant lower precision in SD (*p* = 0.012) for the *y*-component relative to the *x*-component, no significant difference was found between the *x*- and *y*-component of S2S precision (*p* = 0.35).

**Table 2:** The pooled mean precision of the eye-tracker for the horizontal, vertical and vectorial component of the gaze

Precision	Mean_All Stimuli_ ± SD (°^2^)	Mean_Central Stimuli_ ± SD (°^2^)	Mean_Peripheral Stimuli_ ± SD (°^2^)
BCEA	0.51 ± 0.39	0.34 ± 0.30	0.79 ± 0.69
Precision	Mean_All Stimuli_ ± SD (°)	Mean_Central Stimuli_ ± SD (°)	Mean_Peripheral Stimuli_ ± SD (°)
SD_x_	0.23 ± 0.08	0.18 ± 0.07	0.31 ± 0.14
SD_y_	0.30 ± 0.11	0.27 ± 0.12	0.35 ± 0.14
S2S_x_	0.11 ± 0.04	0.09 ± 0.03	0.14 ± 0.07
S2S_y_	0.13 ± 0.05	0.11 ± 0.05	0.14 ± 0.07
S2S_R_	0.18 ± 0.06	0.15 ± 0.06	0.21 ± 0.09

**Fig. 9:**
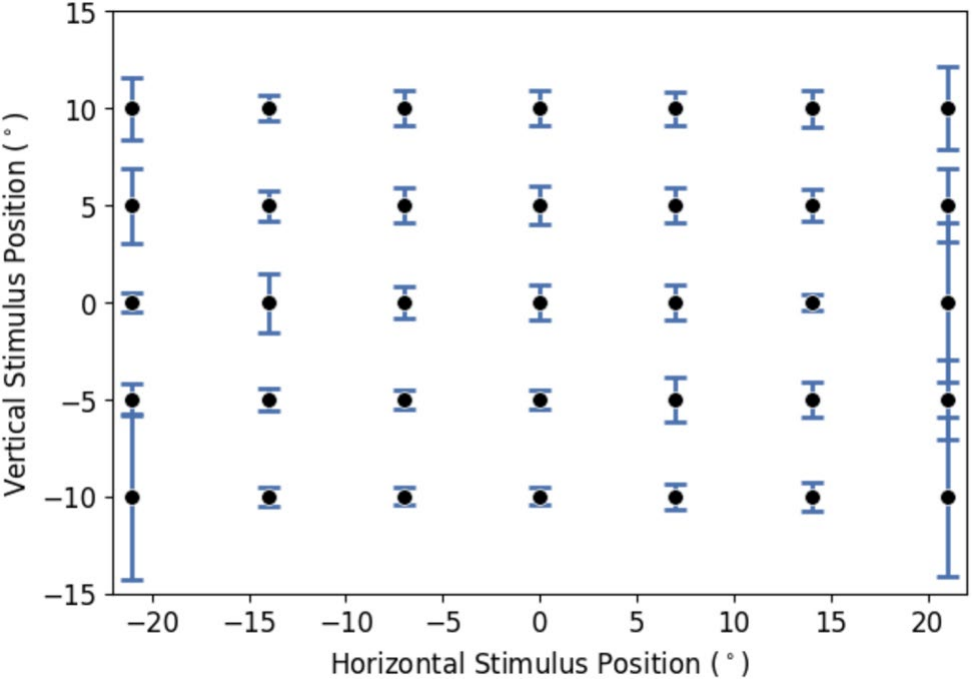
Mean absolute error of vergence for different stimulus locations, expressed with error bars. *Note:* Dots indicate stimulus positions. Error bars indicate the vergence MAE at these locations in degrees

In addition to evaluating the accuracy and precision of gaze fixation, we analyzed vergence error using the same gaze estimates. The estimated vergence errors for all stimulus fixations are shown in Fig. 9. Pooled across all *x* and *y* target locations, we found an average vergence MAE of 1.10 ± 0.95° for all stimuli, 0.68 ± 0.22° for the central stimuli and 2.34 ± 1.37° for the peripheral stimuli. This corresponds to fixation depth estimation error (*z*-axis) ranging from 538 to 821 mm for all stimuli, 576 mm to 746 mm for central stimuli and 450 mm to 1167 mm for peripheral stimuli, assuming an average viewing distance of 650 mm and an interocular distance of 60 mm. The figure shows that the vergence error was relatively constant for the stimuli between – 14° and 14° degrees horizontally, while the error increases for a horizontal angle of – 21° and 21°.

Interestingly, the vergence MAEs in Fig. 9 for fixating targets at horizontal eccentricities of – 14° and 14° are relatively small compared to the horizontal and vertical MAEs of gaze at these locations.

### Experiment 2: Vergence analysis

**Fig. 10:**
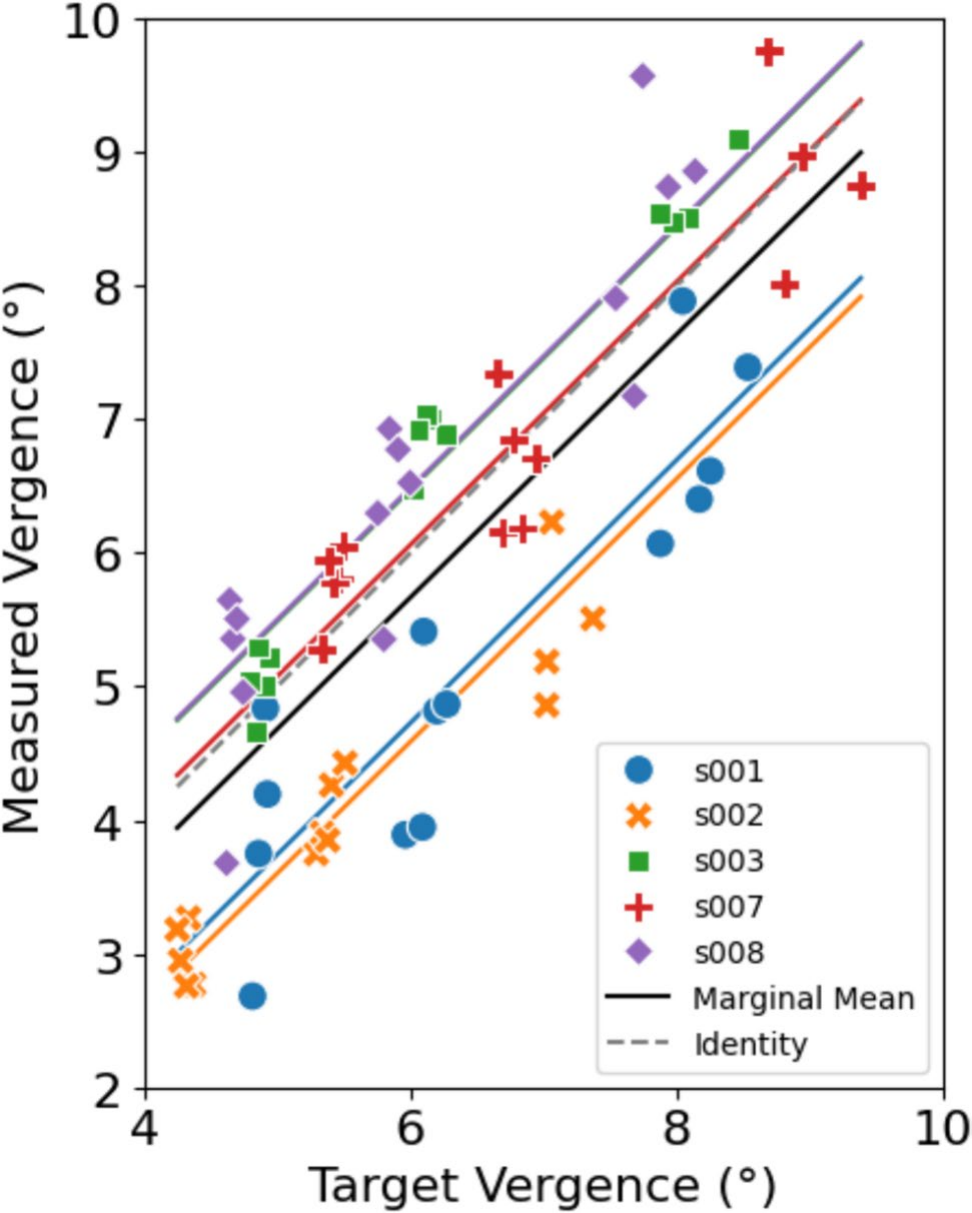
Scatter plot of the target vergence versus the measured vergence. *Note.* The regression line of the linear mixed effects model is plotted, as well as all the individual regression lines for each subject

To further quantify the accuracy and precision of the vergence estimates, we asked participants to fixate targets at different depths. For this part of the second experiment, 5% of the fixation windows were identified as outliers, which we removed from further analysis. The accuracy of vergence estimation, quantified as the average MAE between target and measured vergence, was 0.89 ± 0.58°. However, due to fixation disparity, this MAE measure might underestimate the true accuracy of the vergence estimates. In Fig. 10, we therefore plotted the measured vergence against the target vergence for three different distances for all subjects and fitted a mixed-effect linear regression model to the data. Note that some of the resulting regression lines for the different subjects (colored) fall above and others below the identity line (dashed gray) suggesting that these participants had eso and exo fixation disparity, respectively. The intercept of the model’s fixed effect coefficient was at – 0.25 ± 0.54°, which was not significantly different from zero (*p* = 0.65). The random offset effect (subject) was 0.95 ± 0.56°. The slope of the model’s marginal mean was 0.99 ± 0.05 (*p* = 1.31 × 10^–29^) and the 95% confidence interval ranged from 0.89 to 1.09. Thus, neither the intercept nor the slope were significantly different from that of the identity line.

To account for potential fixation disparities in the measured vergence, we performed an additional analysis in which subject-specific intercepts were subtracted from the measured vergence values. This adjustment assumes that individual fixation disparities introduce a systematic offset in vergence measurements. After correction, the accuracy of vergence estimation improved, with the average vergence MAE decreasing from 0.89 ± 0.58° to 0.49 ± 0.40°. This suggests that a substantial portion of the observed error could be attributed to physiological fixation disparities of the participants rather than true measurement error of the tracking device.

### Experiment 2: Effect of pupil size on vergence estimate

**Fig. 11:**
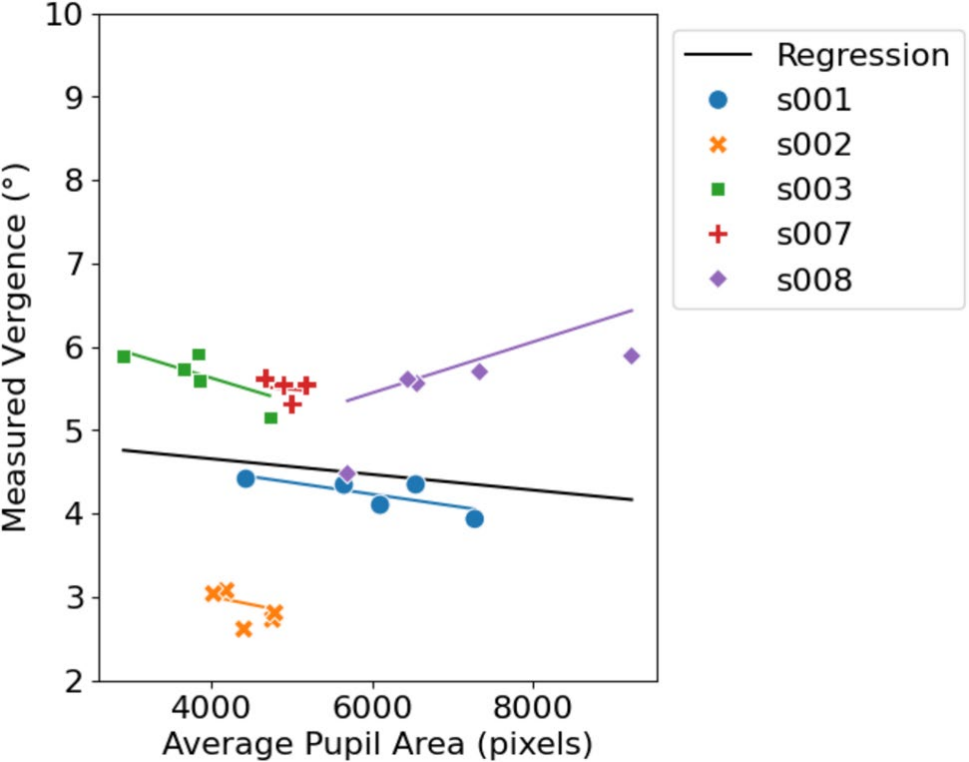
Average projected pupil area in pixels versus the measured vergence. *Note.* Mixed effects linear regression showed no significant influence of pupil area on estimated vergence. A projected pupil area of 5000 pixels, as captured by the camera, corresponded with an approximate pupil diameter of 7 mm

To assess whether vergence estimates with the stereo eye-tracker would be influenced by pupil area, we varied the background illumination of the setup while subjects fixated a stationary target at the center of the screen. All of these fixation epochs yield valid data; no outliers were found. The association between pupil area and vergence is presented in Fig. 11. From this scatter plot, it can be observed that there was no systematic relation between pupil area and measured vergence. The fixed-effect slope coefficient of the linear model was − 9.38 × 10^−5^°/pixels (*p* = 0.53). The Spearman’s rank correlation test showed a correlation coefficient of – 0.026 (*p* = 0.90). Individual Spearman’s rank analysis show that subjects s001 and s003 show a non-significant downward trend (both *r*_s_ = – 0.7, *p* = 0.19). Subject s008 shows high variation of measured vergence (~ 1.5°) with a significant upward trend (*r*_s_ = 0.9, *p* = 0.037).

## Discussion

This study provides a thorough evaluation of the accuracy and precision of a custom-built stereoscopic eye-tracker, focusing on its performance in tracking both gaze and vergence across a wide field of view. The results have important implications for the future of eye-tracking technology, particularly in contexts where binocular vision and vergence information are critical.

### Gaze measurements

The stereo eye-tracker reliably estimated gaze within 1° of the target, particularly for stimuli in the central field of view. This is comparable to other stereo eye-trackers (Barsingerhorn et al., Dec 2018; J. a. T. Chen, Yan & Gray, Wayne & Ji, Qiang, 2008; Shih & Liu Feb, 2004). The accuracy decreased significantly for peripheral targets beyond 14°, with errors increasing to over 1.2° horizontally and 1.4° vertically.

The lower accuracy for the peripheral targets can have several causes. The first involves the misdetection of the correct light source reflections. When a subject fixates on peripheral targets, the glints are on the border of the cornea and occasionally miss the cornea altogether, resulting in multiple glints. This can cause misidentification of glints. The system may wrongly identify a reflection from the sclera or another moist area as a glint, which causes a wrong reconstruction of the corneal center.

Another factor accounting, at least partly, for the decreased accuracy is the shape of the cornea, as is demonstrated in a simulation study by Barsingerhorn et al. (2017). The gaze reconstruction method assumes a spherical cornea, although the cornea is typically aspheric (Navarro et al., 1985). This is especially the case when the glint is close to the cornea-sclera border. Consequently, when the eye is not captured along its optical axis, an error in gaze estimation can be expected of approximately 1° (Barsingerhorn et al., 2017).

These findings highlight the need for enhanced modeling of the eye’s optical properties and the development of advanced algorithms for glint detection and tracking. Another potential solution is to incorporate multiple cameras and/or light sources positioned along the subject’s line of sight. This approach could mitigate the issues related to glint detection and corneal shape distortions. By addressing these challenges, the accuracy of gaze estimates could be significantly improved, making the eye-tracker more versatile and effective across a broader range of applications.

We found a bias in estimating the vertical component of the gaze, particularly when including more horizontally peripheral targets (14° and 21° to both sides). Excluding these peripheral targets reduces the error. For stimuli within – 14° to 14°, the error in vertical gaze estimation was comparable to that of horizontal gaze estimation. The larger error, when including all stimuli, seemed primarily due to the inclusion of peripheral points. For example, the error bars for vertical stimulus positions at 10 degrees in Fig. 4 showed high MAE for peripheral targets, leading to a greater spread for vertical gaze angles.

Precision of the eye-tracker (~ 0.1–0.4°) is similar to the reported precision of commercially available trackers such as the Tobii X2-60 and EyeTribe by Wang et al. Jun (2017). However, the vertical component exhibited lower SD precision compared to the horizontal component. This discrepancy may be attributed to depth estimation errors of *c* and *p*_*v*_ that arise during triangulation. Since the cameras are positioned below the face, these errors are projected onto the vertical component in relation to the screen coordinate system. One potential solution is to position the cameras at eye level, though this could result in the eyes being occluded by eyelashes. Another contributing factor to the reduced vertical precision is the possibility of the eyelid partially occluding the pupil, causing its center to appear lower than its actual position. This misalignment may further impact vertical precision. These factors may also contribute to the slightly lower accuracy observed for the y-component of the gaze.

No significant differences were observed between monocular and binocular viewing conditions, indicating that the eye-tracker is equally effective for both, and the accuracy we report for binocular viewing is marginally affected by fixation disparity. This reliability in binocular measurements supports its use in applications requiring precise vergence analysis.

### Vergence measurements

The accuracy and precision observed under binocular viewing conditions supports its application to measure vergence disorders in humans. The vergence error observed in Experiment 1 tells us that the vergence for the central stimuli, could be estimated with a precision of less than 1°. This is an acceptable accuracy for many applications. Interestingly, the vergence MAE of 0.89° that we found in Experiment 2 was even smaller than the MAE expected if the measurements from the left and right eye were independent. In this case, combining for instance the 0.93° horizontal MAEs for measurements in the central visual field would result in a MAE of about $\sqrt{{0.93}^{2}+{0.93}^{2}}=1.31^\circ$. These promising results have motivated us to further explore the potential of using a stereoscopic eye-tracker to measure vergence.

It is notable that we observed a relatively low MAE in vergence for horizontal gaze angles of – 14° and 14°, despite a reduction in gaze accuracy at these angles. This could be explained by similar error patterns in both eyes. As previously mentioned, the decreased accuracy may result from glint displacement. When the glints shift in the same direction for both eyes, it affects gaze measurement accuracy but does not necessarily impair vergence measurement accuracy.

In the second experiment we further assessed the association between the target vergence and measured vergence. This has shown that a near veridical relationship holds between the target vergence and the measured vergence (fixed-effect slope and bias not significantly different for 1 and 0, respectively). The mixed linear model revealed subject-specific offsets. For some subjects the vergence was systematically underestimated and for others overestimated. This could be due to the subjects’ natural fixation disparity, which is not accounted for by the monocular calibration (Methods). To account for fixation disparity, we further analyzed the accuracy in measuring vergence by adjusting for subject specific offsets, which improved the accuracy by roughly half. However, this additional analysis has limitations, as fixation disparity varies non-linearly with vergence demand (Sheedy, 1980), whereas our offset correction assumes a constant fixation disparity across different demands. This suggests that a substantial portion of the observed error could be attributed to individual fixation biases rather than measurement noise or model inaccuracies.

As concluded above, stereo eye-tracking might work well to measure vergence, but with a vergence accuracy of 0.5° at best. This estimate is based on the measured average accuracy across all subjects, which was found to be 0.49 ± 0.40°. The expected variance reflects variability in measurement accuracy across different subjects and conditions. However, it should be noted that even with this level of accuracy, stereo eye-tracking may not be suitable for use in depth perception tasks, as was previously found by Hooge et al. (2019) for single-camera systems as well. Additionally, fixation disparity, which was not fully accounted for in this study, further limits the accuracy of direct depth estimation from measured vergence values. A vergence error of 1°, for instance, can result in a depth error exceeding 100 mm at a viewing distance of 650 mm with an interocular distance of 60 mm.

### Pupil size

Previous studies have indicated that gaze measurements obtained with video-based eye-tracking methods are systematically influenced by pupil size (Kimmel et al., 2012; Wyatt, 2010). The origin of this pupil-size artifact is still unclear, but it may relate to the projective mismatch between the center of the pupil and the center of the pupil ellipse in the camera’s image, which increases with pupil area (Liebold & Maas, 2024; Wang et al., 1902). Even so, in our second experiment, we did not find a significant association between average pupil area and measured vergence, nor did we find a clear pattern, trend, or correlation. Although individual subjects, such as s001 and s003, visually appear to show a relationship between pupil area and measured vergence, statistical analysis using Spearman rank correlation did not reveal a significant association for any subject. However, s008 did show a significant positive relation between pupil area and measured vergence. Therefore, despite one subject (s008) showing a significant relationship, we conclude that pupil-size variations generally do not substantially impact vergence measurements obtained with a stereoscopic eye-tracker.

However, we note that some subjects (e.g., s002 and s007) exhibited limited pupil size changes, which may have influenced the overall analysis. If pupil size remains nearly constant, any potential effect on vergence measurements might be too small to detect. Excluding these two participants from the analysis also did not yield significant results. Future studies should consider selecting participants with a wider range of pupil size variations to ensure a more comprehensive analysis of this potential effect.

Our results contrast with the findings of Hooge et al. (2019). This may be due to the differences in eye-tracking technology. They used a single-camera eye-tracker that relied on a (nonlinear) mapping function of the pupil-glint vector to estimate gaze, whereas the stereoscopic eye-tracker triangulates the center of corneal curvature and the pupil center to determine the eye’s optical axis and, after adjusting for the angle $\kappa$, the subject’s gaze. Perhaps the use of multiple cameras in the stereoscopic system allows for a more accurate estimation of the pupil center by providing estimates from two different vantage points. To validate this, further research comparing the performance of stereoscopic eye-trackers with those that use mapping functions is needed.

### Limitations

A limitation of the employed eye-tracker setup is the limited 50-Hz frame rate of the cameras’ video feed. Although this suffices for gaze and vergence estimates under static conditions, such a system would not allow, for instance, the study of the fast dynamics of saccades or saccade-vergence interactions (Minken & Gisbergen, 1996; Zee et al., 1992).

Another limitation of our study is that participants were not clinically screened for fixation disparity or subclinical vergence anomalies. While all participants reported normal binocular vision, undiagnosed fixation disparities could have influenced the measured vergence accuracy. Future studies may benefit from incorporating vergence screening tests, such as fixation disparity measurements or near point of convergence assessments, to control for potential inter-subject variability due to vergence function.

## Conclusions

In conclusion, the eye-tracker seems to be well suited to measure gaze in both binocular and monocular viewing conditions. To improve the system and the measurement range of the eye-tracker, further research is needed to find a way to improve glint detection when fixation on eccentric targets is needed. Moreover, vergence measurements from Experiment 2 were accurate (0.49 ± 0.40°) within the tested viewing distance (61–35 cm) and were unaffected by the pupil-size artifact observed in single-camera systems. These findings indicate that the stereoscopic eye-tracker may be particularly well-suited for measuring vergence during binocular viewing; however, it is not suitable for measuring depth perception.

## Supplementary Information

Below is the link to the electronic supplementary material.

Supplementary file1 (DOCX 468 KB)

## Data Availability

The datasets used and/or analyzed during the current study are available at https://doi.org/10.34894/LTATFP.

## Code availability

The custom code used to analyze the data in this study is available at https://github.com/a-pijpaert/Single-eye-tracker-Analysis.git.
