# Supplementary material for: A validation study on the accuracy and precision of gaze and vergence using stereoscopic eye-tracking technology
Source: Behav Res Methods. 2025 Jul 1;57(8):214. doi: 10.3758/s13428-025-02731-1 (PMC12213999; doi:10.3758/s13428-025-02731-1)
Supplement: Supplementary file 1 — Supplementary file1 (DOCX 468 KB) [file 13428_2025_2731_MOESM1_ESM.docx]

**Supplement A**

Screenshot of the Graphical User Interface (GUI).


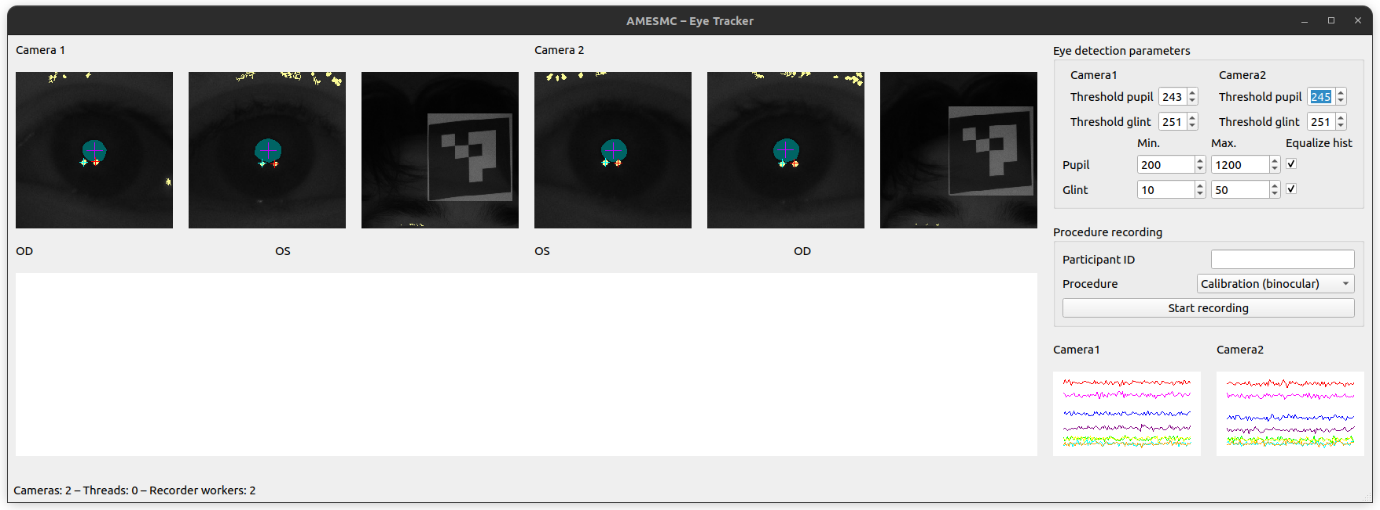
 Note. The region of interest (ROI) of the eye is shown for each eye in each camera, and the ArUco marker ROIs (which were not used in this study) are shown in the left panels. In the right panels, the settings for eye parameter detection can be set up. The lower left corner is used to show the online stimulus location, and the lower right corner shows the pupil glint vectors for each eye in each camera.

**Supplement B**

Flow diagram outlining the process of detecting and processing eye and pupil data.


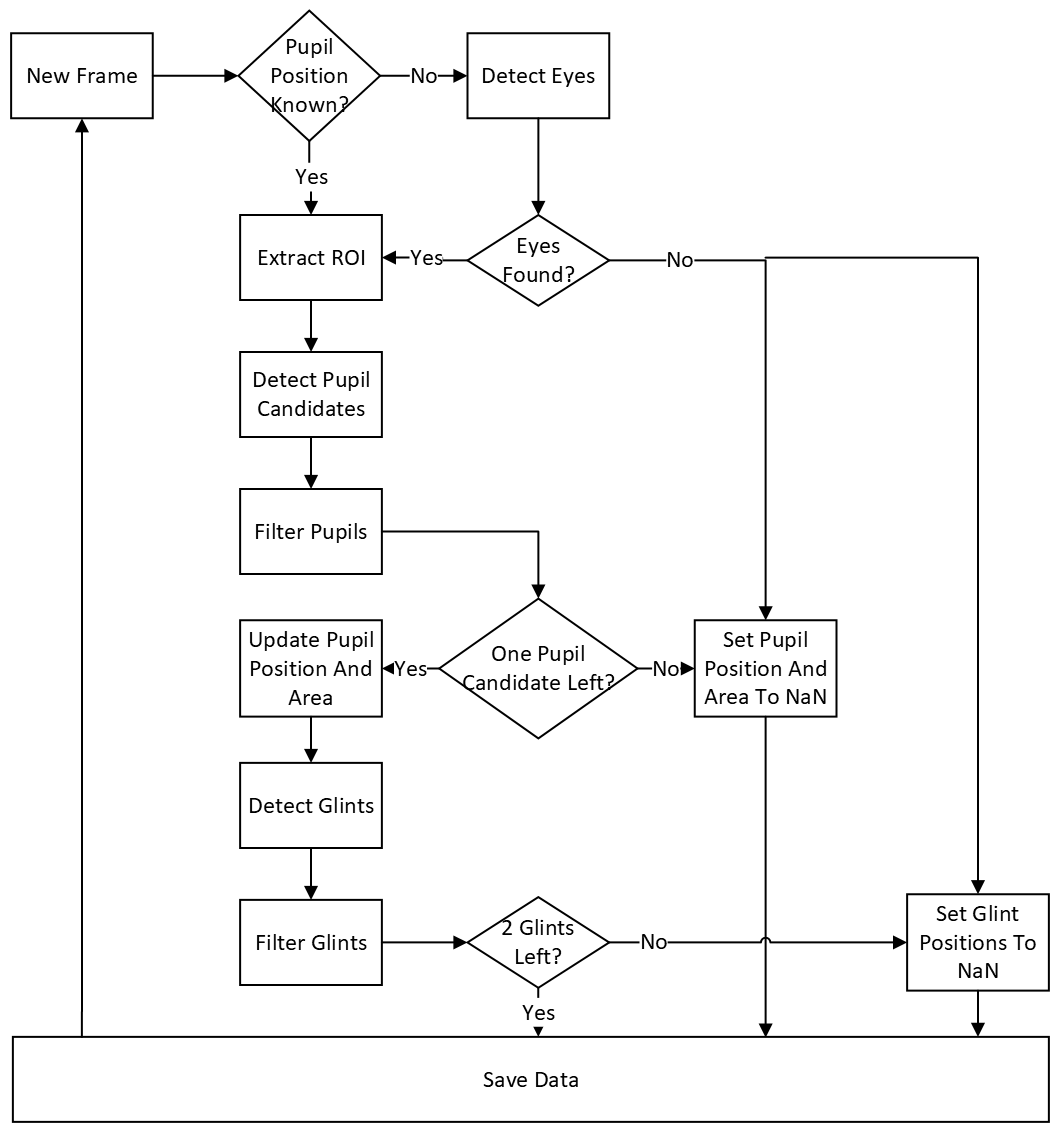


**Supplement C**

3D stimuli used to measure different vergence angles.
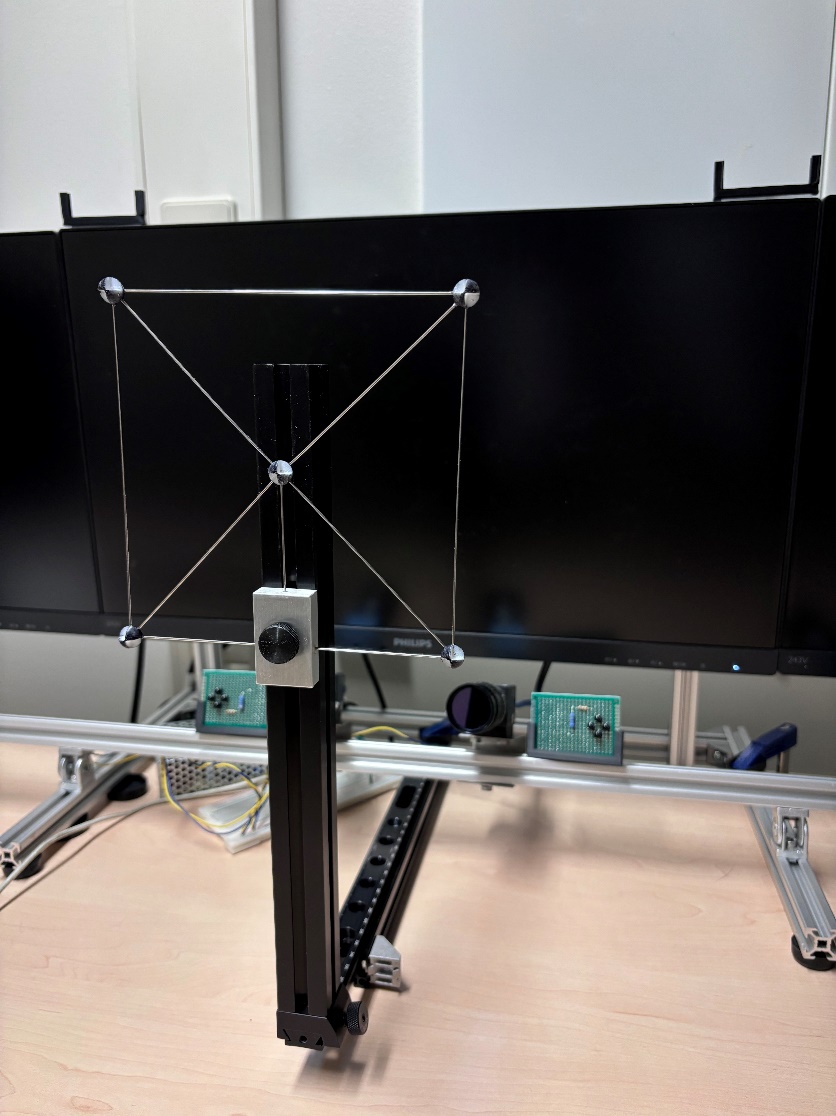


Note. The 3D stimuli were attached to a rail with a rail carrier for easy displacement.
